# Chemometric analysis of ethoxylated polymer products using extracted MALDI-TOF-MS peak distribution features

**DOI:** 10.1371/journal.pone.0328462

**Published:** 2025-07-16

**Authors:** Elizabeth Dickinson, Richard N. Cawthorne, Peter Dodds, Graham M. Atkinson, Pablo F. García Thomas, Lucy Jones, Molly Crosbie, Janet Cook, Julie Wilson

**Affiliations:** 1 Department of Mathematics, University of York, Heslington, York, United Kingdom; 2 Croda Europe Ltd, Goole, East Yorkshire, United Kingdom; Public Library of Science, UNITED KINGDOM OF GREAT BRITAIN AND NORTHERN IRELAND

## Abstract

MALDI-TOF MS (matrix-assisted laser desorption/ionization time-of-flight mass spectrometry) of ethoxylate products produces spectra with distributions of regularly spaced peaks resulting from the addition of monomer units of ethylene oxide to the oligomer. We show that overlapping peak distributions from the different ethoxylated constituents of natural raw materials can be resolved, so that features of the individual distributions (*m/z* at distribution maximum, intensity at the distribution maximum, width of the distribution at half height, and ratio of the distribution to the major peak distribution) can be extracted and used with statistical pattern recognition techniques to study ethoxylated products. Crucially, we weight the extracted features, so that features from a distribution with a high ratio to the main distribution are given more importance (‘ratio-scaled’). We exemplify the method by characterizing the structural variation between types of compositionally diverse Polysorbate 80, PEG castor oil and Oleth-20, and compare the chemometric analysis using our extracted features with analysis of the full spectra. We demonstrate that using ratio-scaled extracted features gives superior results to the full spectrum, both in terms of identifying subtle compositional differences that would otherwise be missed, and in interpretability. Importantly, the integrated auto-assignment of peak distributions to possible compounds allows the results to be reported in terms of the most abundant oligomers of the raw material constituents. This simplification facilitates interpretation of the results and allows the comparison of closely related products.

## Introduction

**Fig 1:**
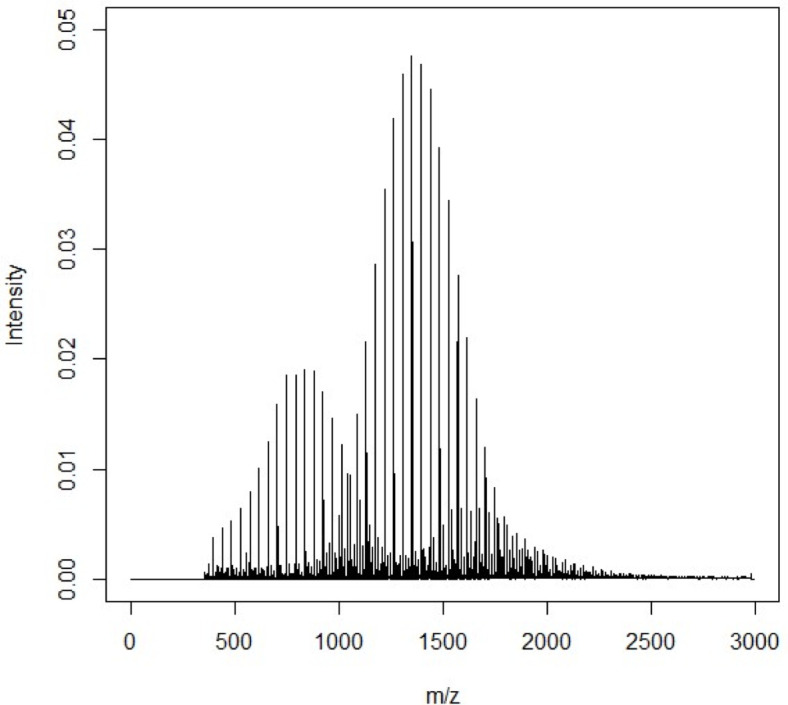
MALDI-TOF-MS spectrum of PEG castor oil. The spectrum shows many regularly spaced peaks, which make up several overlapping distributions from different ethoxylated components.

MALDI-TOF MS (matrix-assisted laser desorption/ ionization time-of-flight mass spectrometry) is a well-known analytical technique that is often used for the study of polymers and peptides [1–3]. Typically generating singly charged ions [4], this soft ionization [3] technology has proven to be important for structural characterisation in industrial chemistry [5]. Ethoxylated products are widely used in the personal care [6,7], pharmaceutical [8,9], food [10,11] and surfactant industries [7,12,13] and they respond exceptionally well to analysis using MALDI-TOF [9,14]. These products are made through living polymerisation with ethylene oxide as the monomer [15,16], ethoxylating natural raw materials such as mixtures of fatty alcohols and acids [7], producing a mixture of ethoxylated constituents within the final product [7,17]. The degree of ethoxylation of these fatty alcohols and acids affects the amphiphilicity of the resulting products [9]. Through calibrated acquisition and optimized matrix selection [18], the addition of monomers to the oligomer can easily be monitored by the presence of regularly spaced peaks in the MALDI spectrum, with a difference of fixed mass of 44 Da between peaks. As shown by Zhu et al. [19], such peaks make up a bell-shaped distribution (Fig 1), where the peak of greatest intensity at the distribution centre relates to the most abundant oligomer present [16]. Depending on the complexity of the product, several overlapping distributions can be present in the spectrum from the various different ethoxylated constituents [17]. This leads to difficulties in comparison between spectra (and therefore products), and the need for multivariate methods, such a principal component analysis (PCA) and partial least squares regression (PLS-R), to analyse such data.

**Fig 2:**
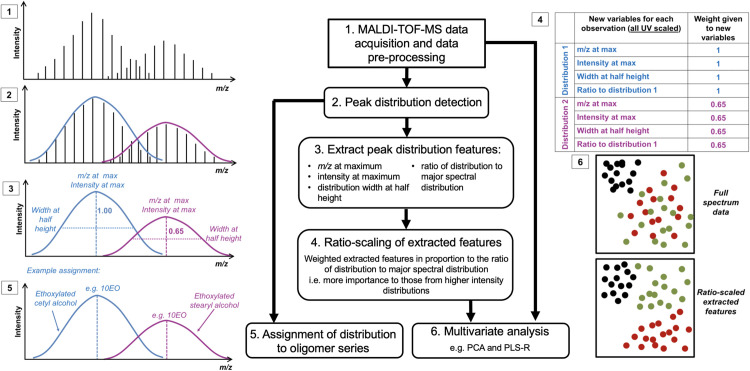
Workflow of methodology. Steps of investigation are shown through two routes, where multivariate analysis of the full MALDI-MS spectrum is compared to analysis of the extracted distribution features.

The use of chemometric techniques to compare samples is a familiar practice in analytical chemistry and the methods can be applied to MALDI spectra of industrial ethoxylates to provide information on the composition of products. However, the identification of multiple peaks from the same distribution is less meaningful when these peaks could be grouped to represent one ethoxylated species of various oligomer lengths in a complex mixture. Commercial packages exist to study polymers, such as Polytools from Bruker [20], which are able to resolve these peak distributions performing Kendrick Mass Defect Analysis to aid comparison of spectra. This method produces visual representations with aligned repetitive patterns found within the polymer, simplifying complexities such as different adducts or charges [21]. However, our work goes further than any known commercial packages or analyses available, developing a multivariate analysis with *weighted* variables based on the ratios of the resolved peak distributions. Here we extract characteristic features of these peak distributions: *m/z* at distribution maximum, intensity at the distribution maximum, width of the distribution at half height, and, most importantly, the ratio of the distribution to the major peak distribution in the spectrum. These new variables of distribution features are weighted by scaling by relative importance, based on the ratio of the minor constituent to the major constituent, as shown in Fig 2. This allows detection of differences that multivariate analysis using the full spectrum would otherwise miss. Furthermore, the known masses of the possible raw material constituents also allow assignment of each distribution to a particular oligomer series. Ratios of such distributions to the main ethoxylated product constituent make the results more meaningful, allowing results to be interpreted in terms of the relative proportions of the compounds present in the product. This makes the results accessible to non-MS specialists and provides a methodology suitable for comparison in industrial polymer manufacture. Such comparisons are essential for quality assurance to ensure that product composition remains consistent for customers, perhaps when the same product may be manufactured at different global locations and is reliant on naturally varying raw materials, or perhaps when manufacture is scaled up or moved between different industrial plants.

## Materials and methods

### Sample selection and preparation

All samples analysed were provided by speciality chemical manufacturer Croda Europe Ltd. Although the same methodology could be applied analogously to a propoxylates, the products selected and discussed in this study are all ethoxylates due to both sample availability and for consistency of product type. These were Polysorbate 80 (used in beauty products), PEG castor oil (used in agricultural and pharmaceutical applications) and Oleth-20 (used in beauty products). The number of batches analysed of the three types of ethoxylates are shown in Table 1. All samples were dissolved in >99.95% methanol (Fisher scientific) to a concentration of 45 mg/mL. The matrix used was α-cyano-4-hydroxycinnamic acid (CCA, 99%, Sigma Aldrich) dissolved in >99.95% methanol to a concentration of 45 mg/mL. The adduct salt used was sodium lactate (NaLac, 99%, Fisher scientific), dissolved in >99.95% methanol to a concentration of 30 mg/mL. 50 μL aliquots of each sample solution were mixed with 50 μL of matrix solution and 50 μL adduct salt solution. This resulted in spotting solutions containing equivalent of 15 mg/mL sample, 15 mg/mL matrix and 10 mg/mL adduct salt. The spotting solutions were spotted onto a 384 well stainless steel MALDI plate using a 10 μL plastic pipette tip. The spots were left to dry at ambient temperature for ~ 1 minute before being inserted into the MALDI-MS instrument for analysis. Samples were spotted onto the MALDI plate in a randomised manor to combat any plate inhomogeneity [22,23]. Five spots were arranged in a diagonal line on the plate to avoid confining sites to one area of the plate, resulting in five technical replicates for each sample. Samples were run in batches of 10 maximum (50 spots).

**Table 1:** Number of batches of ethoxylates analysed.

Product	Type	Number of batches
Polysorbate 80	1	18
	2	6
	3	6
PEG Castor Oil	1	27
	2	17
Oleth-20	R1*	10
	R2*	8
	R3*	20

For each batch of respective ethoxylates, four or five replicates were analysed by MALDI-TOF-MS. *See Table 4 for further information.

### Data acquisition

The instrument used for data acquisition was a Shimadzu Axima Performance MALDI-TOF mass spectrometer. Calibration of the mass spectrometer was achieved using a simple polymer of known masses within the range of product being analysed, e.g., polyethylene glycol (PEG). Prior investigations into optimum matrix showed that α-cyano-4-hydroxycinnamic acid (CCA) performed best across the range of masses under investigation. Pre-runs were used to optimise laser power for each product (generally between 70–100) to prevent fragmentation and maintain spectral output within 50mv-500mV. Any MALDI spectra outside of this range, or with excess noise or evidence of contamination/additional peaks were deemed to be poor quality spectra and either re-run or dis-carded, ensuring that each sample had at least 4 replicates for data analysis. Mass spectra were exported as comma delimited ASCII files for processing using the Shimadzu batch processor program.

### Data pre-processing

Alignment of all processed MALDI-MS spectra was conducted in R version 4.1.1 (R Core Team 2021, R Foundation for Statistical Computing, Vienna, Austria) using the packages MALDIquant and MALDIquantForeign [24]. Using C code written in-house, counts were combined to give data at 0.1 Da resolution in order that spectra from different samples could be compared. This was achieved by adding the counts to the nearest 0.1Da bins above and below the recorded *m/z* value in proportion to their proximity. Matrix peaks were removed by replacing the intensities with the mean average of the intensities for peaks one ethylene oxide (EO) unit away on either side. To improve the consistency between technical replicates, all spectra were normalized to the same total integral before replicate analyses were combined by averaging intensities over the available spectra. The resulting data are referred to as the full MALDI spectra in the following analyses.

### Distribution detection and feature extraction

Again, using C code written in-house, the *m/z* value for the most abundant compound was identified from the maximum intensity in each spectrum, and the common 44 Da spacing used to track the related ions, which corresponded to the distribution of EO units added. After storing the *m/z* values and intensities characterising this compound’s distribution, these intensities were removed from the data matrix so that the next most abundant distribution could be identified. In this way, new distributions were identified until the maximum intensity available was less than a predefined threshold. Similarly, a threshold was used to determine when the intensity of an ion is too low to be considered part of a distribution. As the distributions were identified within each individual spectrum, they needed to be matched across all spectra for comparisons to be made. Each distribution in the first spectrum was given an ID number and, where possible, matched based on *m/z* values to the corresponding distribution in all other spectra. Any distribution occurring in the second spectrum, but not the first, was then assigned the next ID number and the matching repeated in subsequent spectra. This process was repeated until all distributions were associated with an ID number. For each identified distribution, the maximum intensity and its m/z value, as well as the width of the distribution at half height, were recorded for each spectrum the distribution occurred in, providing information on the range and mean number of EO units for the compound. These features, together with the intensity ratio of each distribution to the first distribution, represent the mass spectra and were used as variables in a multivariate analysis.

### Ratio-scaling of extracted features

As the extracted distribution features are on different scales, some form of re-scaling was necessary. Whilst scaling to unit variance (UV) would prevent those with greater values dominating the analysis, this would also give equal weight to every distribution, even those close to the noise level. Therefore, a new scaling method was developed for the extracted distribution features so that more important distributions had greater weight in the analysis. For each distribution, the extracted variables (*m/z* at distribution maximum, intensity at the distribution maximum, width of the distribution at half height, and ratio of the distribution to the major peak distribution) were UV-scaled and then were subsequently weighted in proportion to the ratio between the average intensity for this distribution and the average intensity for the first major distribution (Fig 2). These ratios used for weighting were calculated before the variables were UV-scaled. This resulted in the variables for a particular distribution having equal weight but gave more importance to those from higher intensity distributions. The resulting data are referred to as the extracted features in following analyses.

### Multivariate analysis

Principal component analysis (PCA) was performed as an unsupervised method to determine any patterns or outliers, followed by supervised analysis using partial least squares regression (PLS-R) for classification, all conducted in R version 4.1.1. The pls package was used for PLS-R [25]. For the classification of different types of PEG castor oil, spectra were classified by taking predicted values less than 1.5 as Type 1 and those above 1.5 as Type 2. 3-fold cross-validation was used to obtain more accurate error estimates.

### Compound identification

Using knowledge of the chemical reactions taking place during manufacture and the composition of the raw materials, lists of possible constituents with associated *m/z* values were generated using in-house R scripts; such constituents and associated masses were generated from the synthesis of all possible intermediates before final product synthesis. These masses were subsequently matched automatically to the masses found in the spectral distributions assigned to the ethoxylated products – this was possible through accurate masses generated through effective instrument calibration. At this resolution, the distributions for the second and even third isotopes of a compound can be identified separately, but this is accounted for in the compound identification.

The comparisons between these constituents of products analysed are all relative proportions and qualitative – ionization efficiencies based on hydrophobicity/hydrophilicity balance of the components are therefore not affecting the reported comparisons. No calibration with standards was carried out for quantitative absolute concentrations, as this was not necessary for the comparisons or the purpose of the investigation.

## Results and discussion

**Fig 3:**
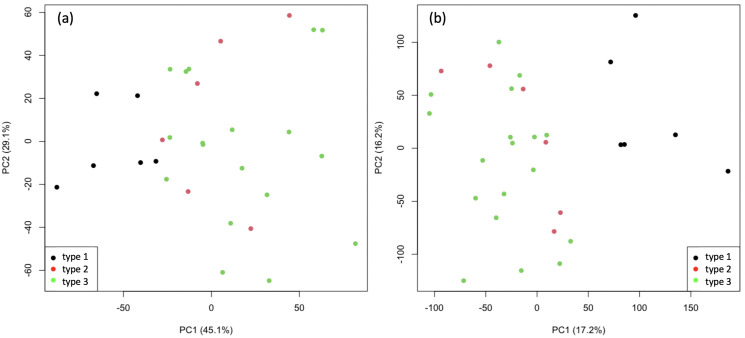
PCA score plots of MALDI data of polysorbate 80. Scores plot for the first two principal components obtained from full MALDI spectra unscaled (a) and scaled (b) of three different types of Polysorbate 80, showing that types 2 and 3 are indistinguishable. Observations are coloured by type.

**Fig 4:**
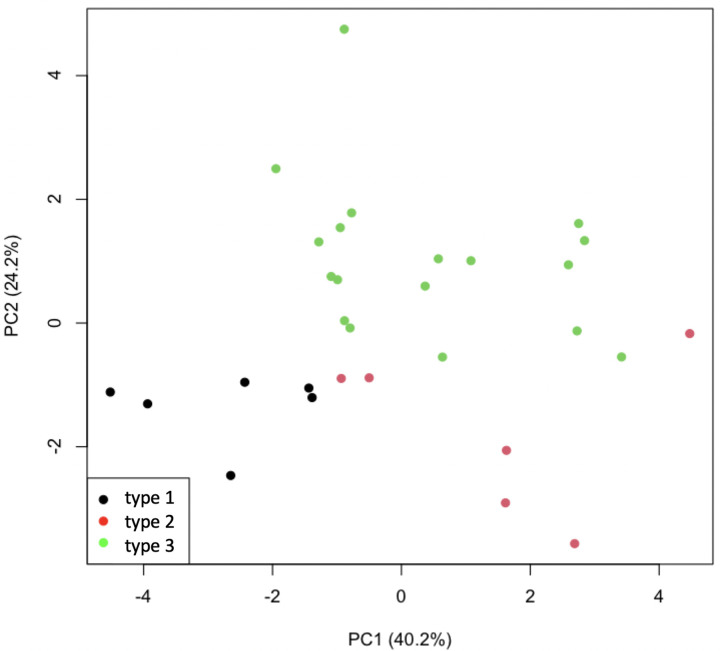
PCA score plots of extracted distribution features of MALDI data of polysorbate 80. Scores plot for the first two principal components obtained from features extracted from the distributions of peaks identified in the mass spectra of Polysorbate 80. The plot shows that there is distinction between all types of Polysorbate 80. Observations are coloured by type.

Polysorbate 80 is comprised of oleate esters, sorbitol and sorbitol anhydrides condensed with approximately 20 moles of EO, but different types of this product may vary in composition, for example by subtle fluctuating natural raw material composition. Although compositional differences were observed between PS80 Type 1 and the other types when using the full data (that is, all peaks in the mass spectra), it was not possible to distinguish between the other Types 2 and 3 from MALDI data alone. This was the case whether the data is unscaled (Fig 3a) or scaled to unit variance to prevent large peaks dominating the analysis (Fig 3b). The separation was greater for scaled data showing that smaller peaks were contributing to the difference in PS80 Type 1 samples. This was especially observed in the main peak distribution, most likely from the ethoxylation of Sorbitan/Sorbitan monooleate/Sorbitan dioleate/Isosorbide monopalmitate – many possible ethoxylated species with the same mass made identification of individual constituent oligomer series impossible. Type 1 had the lowest degree of ethoxylation with 2–3 EO units less than the other types. However, the PCA scores plot in Fig 4 showed that the analysis of features extracted from each identified distribution not only separated Type 1 samples along the first principal component (PC1) but also distinguished between Type 2 and Type 3 along the second component (PC2). Type 3 could be differentiated from the other PS80 types based on the presence of ethoxylated high molecular weight constituents, including PEG sorbitol. This demonstrates how the chemometric analysis of extracted MALDI-MS distribution features is superior in identifying subtle compositional differences over and above the analysis of the full spectrum.

**Fig 5:**
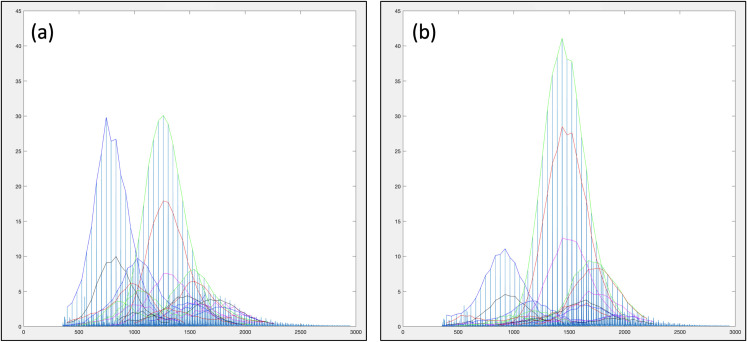
Distributions isolated within the MALDI-MS spectrum of a typical batch of PEG castor oil. Plots relate to (a) type 1 and (b) type 2.

**Fig 6:**
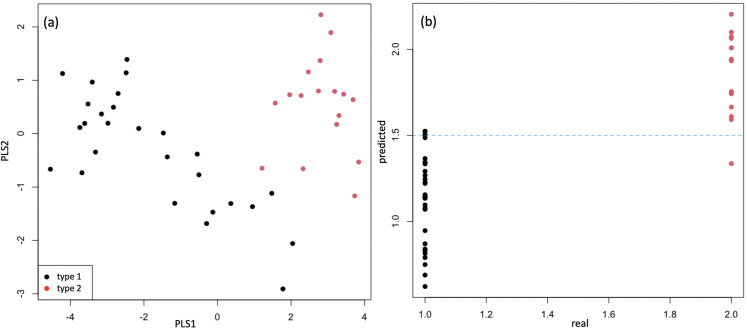
Partial least squares regression (PLS-R) analysis of PEG Castor oil using extracted features of MALDI-MS data. The scores plot for the first two PLS latent variables is shown in (a) with the values predicted by the PLS-R model using 3-fold cross-validation in (b). Here Type 1 and Type 2 are encoded as 1.0 and 2.0 respectively. The dotted line represents a predicted value of 1.5, taken as the boundary between classes.

**Fig 7:**
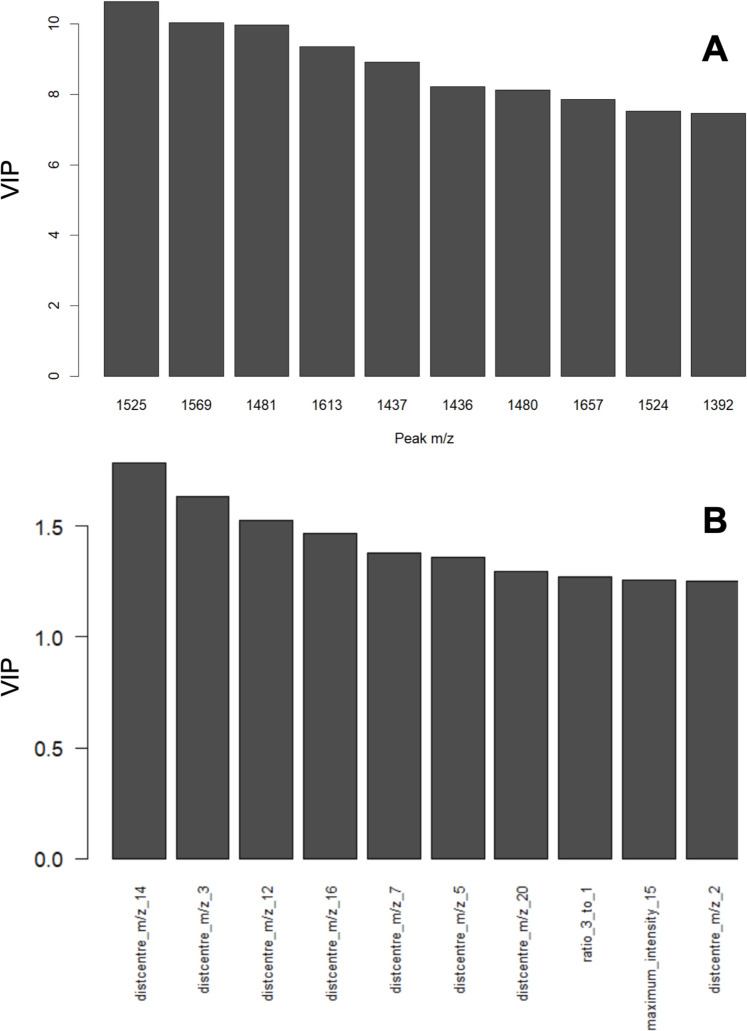
Variable importance in projection (VIP) plots from PLS-R of PEG Castor oil MALDI data. (A) Full spectrum analysis, showing important variables as MALDI peaks (m/z); (B) Extracted features analysis showing more informative results, based on general distribution shifts (distribution maxima m/z) and ratios of distributions and therefore constituents.

Fig 5 shows typical MALDI spectra for each type of PEG castor oil product, with the peak distributions isolated. It can be seen that the ratios of main compounds vary between the two types, although some expected batch-to-batch variance can occur within each type (not shown). Using the *m/z* value of the maximum intensity, these distributions can be assigned (within 0.5 *m/z* units) to sodiated PEG with an average 18 EO units (*m/z* 834) and sodiated glycerol with an average 29 EO units (*m/z* 1392). The ratio of glycerol to PEG is increased in Type 2 in relation to Type 1. This difference can also be seen in partial least squares regression (PLS-R) analysis of the extracted distribution features (Figs 6 and 7, and Tables 2 and 3). Fig 6a shows the scores plot obtained from this supervised method that relates the variance in the variables to a response (or responses), in this case the type, encoded as 1.0 for Type 1 and 2.0 for Type 2. The results shown in Table 2 are those obtained for test data that was not used during training. It is clear that PLS-R using extracted features produces a successful model with 95% classification rate. In comparison, PLS-R using the full spectrum produces a less successful model with 59% correct classification rate (Table 3), where Type 1 is often classified as Type 2. However, as well as model performance, the purpose of this analysis is to show that the variable importance in projection (VIP) of the full spectrum analysis are difficult to interpret, stating importance of individual MALDI-MS peaks, and are therefore not particularly useful without subsequent further investigation and prior MS knowledge. This is shown in plot A of Fig 7. Conversely, the VIP of the extracted distribution features gives easily interpretable results, even for the non-MS specialist, as shown in Fig 7 plot B. This plot is more informative, showing that for several distributions, the *m/z* at distribution maximum is clearly important in distinguishing between the two types of product, most likely signifying a shift of the distribution between the types of PEG castor oil, and a subtle different level of ethoxylation between the two types of product. Similarly, the ratio of distributions from PEG and glycerol (‘ratio 3 to 1’) has also been shown to be important in plot B, demonstrating how the proportions of constituents can be highlighted for product classification, and ultimately quality control.

**Table 2:** PLS classification results of test data for PEG castor oil using extracted distribution features.

		Predicted Class	
		Type 1	Type 2
Real Class	Type 1	26	1
	Type 2	1	16

Successful classification rate is 95%. Values were obtained by taking predicted values less than 1.5 as Type 1 and those above 1.5 as Type 2.

**Table 3:** PLS classification results of test data for PEG castor oil using the full spectrum.

		Predicted Class	
		Type 1	Type 2
Real Class	Type 1	9	18
	Type 2	0	17

Successful classification rate is 59%. Values were obtained by taking predicted values less than 1.5 as Type 1 and those above 1.5 as Type 2.

**Fig 8:**
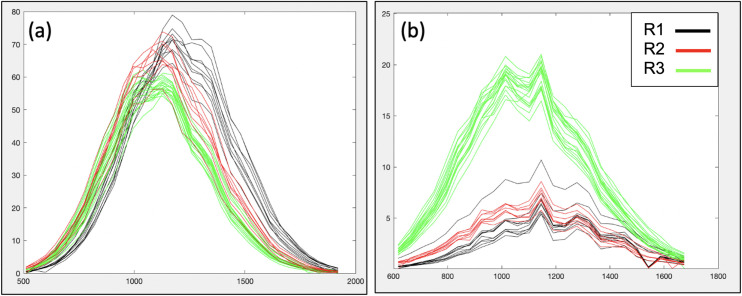
Distributions obtained from the peak maxima for the two highest intensity ethoxylated constituents of Oleth-20, coloured according to type. (a) shows the distribution attributed to ethoxylated oleyl alcohol and (b) that assigned to ethoxylated cetyl alcohol.

Finally we were able to demonstrate the method’s ability to identify and, more importantly, show the impact of differences in the ethoxylation process using samples of Oleth-20. As shown in Table 4, type R1 of Oleth-20 has a higher ethylene oxide to fatty alcohol ratio than the other two types. The impact of this can be seen clearly in Fig 8a when only the curves obtained by connecting the peaks within a distribution are plotted, with one curve for each observation. For type R1, this resulted in a shift of the main distribution to higher mass (corresponding to ~ 2 EO units) compared to the other two types R2 and R3. This distribution was assigned to the highest intensity/concentration constituent, ethoxylated oleyl alcohol, based on molecular weight. Fig 8b shows that type R3 observations have very different intensities from R1 and R2 for the distribution assigned as ethoxylated cetyl alcohol (with the order reversed from that seen for oleyl alcohol in Fig 8a). This can be explained by the different percentages of the major fatty alcohol used in the process (Table 4). This type of presentation of the data, and auto-assignment of extracted distributions to chemical constituents, is easy to summarise and interpret, so that the impact of process changes can easily be monitored. This would be very difficult to accomplish and visualise using the full MALDI-MS spectrum, due to obvious complexity of many peaks.

**Table 4:** Types of Oleth-20 analysed.

Type	EO to fatty alcohol ratio	Major fatty alcohol %
R1	3.35	90–98
R2	3.01	90–98
R3	2.91	70–90

Differences in the ethoxylation process are shown between types R1, R2 and R3 of Oleth-20.

## Conclusions

In all example analyses, identification and isolation of the peak distributions relating to particular constituents not only reduced background noise and simplified interpretation of the results, but also provided the potential for compound identification. Subtle differences between types can be distinguished and interpreted more easily using ratio-scaled distribution features in comparison to use of the full spectrum. Although the three examples used for demonstration are all ethoxylated species, the methods can easily be transferred to propoxylated products, which would follow the same methodology but with a greater spacing of 58 Da between peaks (molecular mass of propylene oxide) in a distribution rather than the 44 Da observed in ethoxylates. In future, the process could be extended to include the deconvolution of overlapping peak distributions using information on the expected isotope distributions of particular isobaric compounds.

Despite the fact that MALDI-TOF is a soft ionization technique with minimal fragmentation [3], any fragments produced would occur at the same molecular weight as the low EO constituents and be difficult to differentiate. This fragmentation, however, would still not impact the overall concept and methodology when making comparisons between types of polymer products or identifying and assigning the distributions to ethoxylated species in a mixture – the same ethoxylated species found in products with subtle differences in concentrations would undergo the same fragmentation, therefore the methodology described here would be unaffected, and still be applicable and useful.

Although we have given three different examples to demonstrate the success of this new methodology, this has proven successful across many more products throughout our investigation and has also been extended to compare similar products between different manufacturers, to extrapolate confidential competitor manufacturing processes. We have also used the same methods to deduce the impact of new manufacturing conditions on the composition of products, and to test whether raw materials came from sustainable sources.

Most importantly, our method simplifies analysis by providing results that are easily interpreted. Rather than simply reporting changes in peak intensities for different *m/z* values which would need further interpretation, the *ratios* of *identified* constituents are provided and can be reported quickly, for example, as “the greatest variance between two types of product is due to the ratio of distribution 2 (constituent y) to distribution 1 (constituent x)”. This makes the results of analyses more informative and accessible to the wider industrial community, allowing faster, more revealing results to be used for essential decision making. Equally, we demonstrate the method’s simplicity, versatility and ease of application for spectroscopists to adopt. The method could prove to be important in industrial process development, providing assurance that the use of new sustainable technologies does not materially alter the composition of products, ensuring no change to quality and continued consistency of manufacture, thereby maintaining customer trust.

## References

[1] Montaudo G, Samperi F, Montaudo MS. Characterization of synthetic polymers by MALDI-MS. Prog Polym Sci. 2006;31:277–357.

[2] Hickinbotham S, Fiddyment S, Stinson TL, Collins MJ. How to get your goat: automated identification of species from MALDI-ToF spectra. Bioinformatics. 2020;36(12):3719–25. doi: 10.1093/bioinformatics/btaa181 32176274 PMC7320604

[3] Fouquet TNJ, Cody RB, Charles L. Degradation strategies for structural characterization of insoluble synthetic polymers by mass spectrometry. Mass Spectrom Rev. 2024;:10.1002/mas.21903. doi: 10.1002/mas.21903 39093552 PMC12502030

[4] Mao J, Zhang B, Zhang H, Elupula R, Grayson SM, Wesdemiotis C. Elucidating Branching Topology and Branch Lengths in Star-Branched Polymers by Tandem Mass Spectrometry. J Am Soc Mass Spectrom. 2019;30(10):1981–91. doi: 10.1007/s13361-019-02260-0 31363988

[5] Lee D, Rumbelow S, Williams SKR. Identification and quantitation of trace impurities in fatty alcohol ethoxylates using HPLC and MALDI-TOF mass spectrometry. Anal Chim Acta. 2009;654(1):59–63. doi: 10.1016/j.aca.2009.04.032 19850169

[6] Bhargava HN, Leonard PA. Triclosan: applications and safety. Am J Infect Control. 1996;24(3):209–18. doi: 10.1016/s0196-6553(96)90017-6 8807001

[7] Lukosek M, Emmons-Burzyńska M, Alejski K, Szwach I. Physicochemical Characterization of Ethoxylation Products of Fatty Acid Esters. Front Chem Eng. 2021;3. doi: 10.3389/fceng.2021.617701

[8] Wuchner K, Yi L, Chery C, Nikels F, Junge F, Crotts G, et al. Industry Perspective on the use and Characterization of Polysorbates for Biopharmaceutical Products Part 1: Survey Report on Current State and Common Practices for Handling and Control of Polysorbates. J Pharm Sci. 2022;111(5):1280–91. doi: 10.1016/j.xphs.2022.02.009 35192858

[9] Raith K, Schmelzer CEH, Neubert RHH. Towards a molecular characterization of pharmaceutical excipients: mass spectrometric studies of ethoxylated surfactants. Int J Pharm. 2006;319(1–2):1–12. doi: 10.1016/j.ijpharm.2006.03.030 16730931

[10] Frison-Norrie S, Sporns P. Investigating the molecular heterogeneity of polysorbate emulsifiers by MALDI-TOF MS. J Agric Food Chem. 2001;49(7):3335–40. doi: 10.1021/jf010096w 11453772

[11] Ayorinde FO, Gelain SV, Johnson JH Jr, Wan LW. Analysis of some commercial polysorbate formulations using matrix-assisted laser desorption/ionization time-of-flight mass spectrometry. Rapid Commun Mass Spectrom. 2000;14(22):2116–24. doi: 10.1002/1097-0231(20001130)14:22<2116::AID-RCM142>3.0.CO;2-1 11114018

[12] Tesser R, Russo V, Santacesaria E, Hreczuch W, Di Serio M. Alkoxylation for Surfactant Productions: Toward the Continuous Reactors. Front Chem Eng. 2020;2:7.

[13] Pascale R, Acquavia MA, Onzo A, Cataldi TRI, Calvano CD, Bianco G. Analysis of surfactants by mass spectrometry: Coming to grips with their diversity. Mass Spectrom Rev. 2023;42(5):1557–88. doi: 10.1002/mas.21735 34570373

[14] Penfield KW, Rumbelow S. Challenges in polysorbate characterization by mass spectrometry. Rapid Commun Mass Spectrom. 2020;34 Suppl 2:e8709. doi: 10.1002/rcm.8709 31943438

[15] Grubbs RB, Grubbs RH. 50th Anniversary Perspective: Living Polymerization—Emphasizing the Molecule in Macromolecules. Macromolecules. 2017;50(18):6979–97. doi: 10.1021/acs.macromol.7b01440

[16] Li Y, Zhou J, Zhang Y, Liang H, Sun J, Liu Y, et al. Synthesis and Properties of Primary Alcohol Ethoxylates Using Different Catalytic Systems. ACS Omega. 2021;6(44):29774–80. doi: 10.1021/acsomega.1c04232 34778650 PMC8582037

[17] Di Serio M, Di Martino S, Santacesaria E. Kinetics of Fatty Acids Polyethoxylation. Ind Eng Chem Res. 1994;33(3):509–14. doi: 10.1021/ie00027a006

[18] Borisov RS, Polovkov NY, Zhilyaev DI, Zaikin VG. Matrix effect in matrix-assisted laser desorption/ionization mass spectra of derivatized oligomeric polyols. Rapid Commun Mass Spectrom. 2013;27(2):333–8. doi: 10.1002/rcm.6449 23239381

[19] Zhu H, Yalcin T, Li L. Analysis of the accuracy of determining average molecular weights of narrow polydispersity polymers by matrix-assisted laser desorption ionization time-of-flight mass spectrometry. J Am Soc Mass Spectrom. 1998;9(4):275–81. doi: 10.1016/S1044-0305(97)00292-4 9879362

[20] Available from: https://www.bruker.com/en/products-and-solutions/mass-spectrometry/ms-software/polytools.html

[21] Fouquet TNJ. The Kendrick analysis for polymer mass spectrometry. J Mass Spectrom. 2019;54(12):933–47. doi: 10.1002/jms.4480 31758605

[22] Hajduk J, Matysiak J, Kokot ZJ. Challenges in biomarker discovery with MALDI-TOF MS. Clin Chim Acta. 2016;458:84–98. doi: 10.1016/j.cca.2016.04.033 27134187

[23] Malys BJ, Piotrowski ML, Owens KG. Diagnosing and Correcting Mass Accuracy and Signal Intensity Error Due to Initial Ion Position Variations in a MALDI TOFMS. J Am Soc Mass Spectrom. 2018;29(2):422–34. doi: 10.1007/s13361-017-1845-2 29218450

[24] Gibb S, Strimmer K. MALDIquant: a versatile R package for the analysis of mass spectrometry data. Bioinformatics. 2012;28(17):2270–1. doi: 10.1093/bioinformatics/bts447 22796955

[25] Mevik BH, Wehrens R. The pls package: principal component and partial least squares regression in R. J Stat Softw. 2007;18:1–23.

