# Supplementary material for: Chemometric analysis of ethoxylated polymer products using extracted MALDI-TOF-MS peak distribution features
Source: PLoS One. 2025 Jul 16;20(7):e0328462. doi: 10.1371/journal.pone.0328462 (PMC12266461; doi:10.1371/journal.pone.0328462)
Supplement: Attachment — Submitted filename: Plos One review 1.docx [file pone.0328462.s001.docx]

This study developed a multivariate analysis with weighted variables based on the ratios of the resolved peak distributions, to aid in the analysis of ethoxylated products using MALDI-TOF MS. Their chemometric analysis of extracted MALDI-MS distribution features showed superior performance in identifying subtle compositional differences over and above the analysis of the full spectrum. The technical quality of the paper is assured, with a lot of data generated, and the presentation is clear. The methodology and analysis look proper and valid. Overall, the study is scientifically sound. Acceptance is recommended after minor revision.

1. In source fragmentation can occur during the ionization process in MALDI-TOF. How did the author address this issue and differentiate it from the actual analyte? I have certain doubts that the observed compounds with low EO counts were merely the ISF of high-EO ones.
2. What’s the detection limits of your targeted analyte using your methods? Is the workflow robust enough to guarantee both sensitivity and specificity good enough for correct identification? I don’t doubt whether they can be found in the manufacturer samples, just curious if it is possible to be extrapolated to other areas such as environmental samples or human specimens.
3. The author claimed that their method can aid in the accurate compound identification of ethoxylated products. I wonder if they can present some evidence as figures, such as the spectra alignment of the identified ones in comparison with the standard chemical to support the statement.
4. Were there any novel ethoxylated chemicals observed in your data mining process, which cannot be matched with any available reference? If so, it adds more value to your infrastructure as for it has the potential to identify new compounds, not only limited to the ethoxylated chemicals but can also be extrapolated to other “modificomics” analysis using MALDI-MS.
5. How will your established workflow benefit the decision-making process in a socioeconomic framework? Please briefly discuss about the reality importance and human relevance in it.
6. Line 224, “ionisation” should be “ionization”.
