# Supplementary material for: Chemometric analysis of ethoxylated polymer products using extracted MALDI-TOF-MS peak distribution features
Source: PLoS One. 2025 Jul 16;20(7):e0328462. doi: 10.1371/journal.pone.0328462 (PMC12266461; doi:10.1371/journal.pone.0328462)
Supplement: Attachment — Submitted filename: Response_to_Reviewers_auresp_2.docx [file pone.0328462.s004.docx]

In response to Reviewer 3's comments, we have now simplified Table 1 by merging cells and only providing the name of each product once.

For the second comment, we apologise for misunderstanding the original comment. We appreciate that the lack of mass spectrometry knowledge required to interpret the results is more relevant to industry than academia. In the abstract, the sentence

"As expertise in mass spectrometry is not required to interpret the results, the method has the potential to aid comparison of closely related products, for example, made at different geographical locations or in different reactors, therefore supporting quality assurance and improving ‘right first time’ rates."

has been replace with

"This simplification facilitates interpretation of the results and allows the comparison of closely related products."

However, we do think that the relevance to industry is important and so, in the introduction, we have left the sentence

"This makes the results accessible to non-MS specialists and provides a methodology suitable for comparison in industrial polymer manufacture. Such comparisons are essential for quality assurance to ensure that product composition remains consistent for customers, perhaps when the same product may be manufactured at different global locations and is reliant on naturally varying raw materials, or perhaps when manufacture is scaled up or moved between different industrial plants."

In the conclusions, we have replaced

"Most importantly, our method provides results that are easily interpreted and can be understood without the need for mass-spectrometry expertise."

with

"Most importantly, our method simplifies analysis by providing results that are easily interpreted."
