# Supplementary material for: Chemometric analysis of ethoxylated polymer products using extracted MALDI-TOF-MS peak distribution features
Source: PLoS One. 2025 Jul 16;20(7):e0328462. doi: 10.1371/journal.pone.0328462 (PMC12266461; doi:10.1371/journal.pone.0328462)
Supplement: Attachment — Submitted filename: Response_to_Reviewers_auresp_3.docx [file pone.0328462.s005.docx]

Thank you for submitting your manuscript to PLOS ONE. After careful consideration, we feel that it has merit but does not fully meet PLOS ONE’s publication criteria as it currently stands. Therefore, we invite you to submit a revised version of the manuscript that addresses the points raised during the review process.

Your manuscript has been evaluated by Reviewers 2 and 3 from the previous round of peer review. Both reviewers are satisfied with your revisions and recommend publication.

No other revisions are required, and I do not anticipate that further review will be necessary.

As the reviewers are satisfied with our revisions and recommend publication as is, we have made no further changes to the manuscript, so that the file labeled 'Revised Manuscript with Track Changes' is now the same as the file labeled 'Manuscript'.

Before we can proceed, we note that your manuscript reports the use of C and R code in the methodology. Please note that PLOS ONE has specific guidelines on code sharing for submissions in which author-generated code underpins the findings in the manuscript. In these cases, we expect all author-generated code to be made available without restrictions upon publication of the work. Please review our guidelines at <https://journals.plos.org/plosone/s/materials-and-software-sharing#loc-sharing-code> and ensure that your code is shared in a way that follows best practice and facilitates reproducibility and reuse.

We have now made our C and R code available together with all data files via the Open Science Framework (OSF) database (DOI 10.17605/OSF.IO/M46T5) and the data availability statement now reads "All data files together with C and R code for data processing are available from the Open Science Framework (OSF) database (DOI 10.17605/OSF.IO/M46T5)."
